# Congenital melanocytic neoplasms: clinical, histopathological and recent molecular developments

**DOI:** 10.1007/s00428-024-04011-3

**Published:** 2025-01-15

**Authors:** Claudia Maria Salgado, Alejandra Tomás-Velázquez, Miguel Reyes-Múgica

**Affiliations:** 1 Division of Pediatric and Perinatal Pathology/Department of Pathology and Laboratory Medicine, Jackson Memorial Hospital Children’s Holtz, University of Miami Miller School of Medicine, 1611 NW 12 Ave., Suite 2153, Miami, FL 33136 USA; 2 Department of Dermatology, University Clinic of Navarra, Madrid, Spain

**Keywords:** Congenital nevus, Neurocutaneous melanocytosis, Pediatric melanoma, Pediatric melanocytic neoplasm

## Introduction

**Fig. 1:**
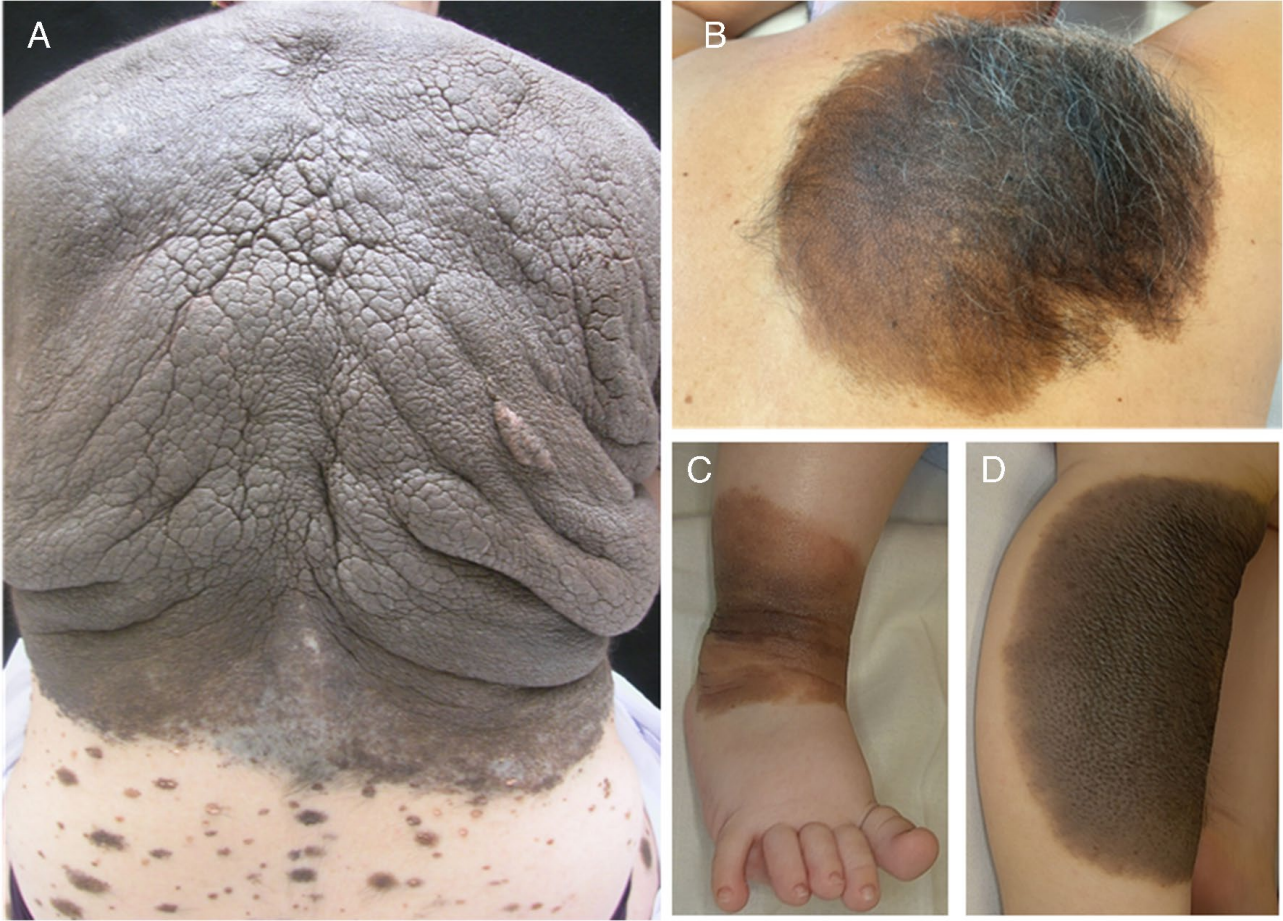
Clinical figures of patients with CMN with different phenotypes. **A** Woman with CMN with bolero pattern, occupying the entire upper and middle back region. Note the folds and grooves with redundant tissue, as well as numerous satellite lesions. **B** Male with CMN located at the right scapular region with marked hypertrichosis. **C**, **D** Two children with CMN on the legs are shown, the first with a localized lesion on the ankle with areas of different color and thickness and the second with a uniform brown lesion of moderate thickness

Congenital melanocytic nevi (CMN) are benign melanocytic neoplasms that develop in utero, becoming evident at birth or within the first months or years of life [1]. Traditionally, they have been classified based on their projected adult size: < 1.5 cm small; 1.5–20 cm medium; and > 20 cm large or giant (PAS) [2]. The prevalence of CMNs varies between 0.2 and 6%, depending on the population studied. The incidence of single small CMNs in newborns is around 1–2%. In contrast, large and giant CMNs are considered a rare disease, with a prevalence of 1/20,000 and 1/50,000–500,000 births, respectively, becoming less frequent as the affected body surface area increases [3–5]. Clinically, in addition to their size, they are characterized based on their color, roughness, nodularity, hypertrichosis, number of satellite lesions [2], and distribution pattern [6]. They can also be associated with alterations in the involved tissue and underlying the lesion, such as atrophy or thickening [7–11], and more importantly, with nevomelanocytic proliferations in the CNS (neurocutaneous melanocytosis, NCM) [12] or malignancy (either of the primary lesion, satellite lesions, or CNS lesions). Thus, their phenotype and progression are highly variable: some lesions remain stable throughout life; a small percentage regress [13]; and others increase in number, proliferate becoming exophytic [7, 8, 14], or present complications such as pruritus [15, 16], progression of NCM, or malignancy (Fig. 1).

NCM is the most frequent complication of multiple, large, and giant CMNs (prevalence ∼25%) and it has been associated with a larger size of the primary nevus, and with a higher total number of lesions. Symptomatology depends on the location and extent of the lesions (intraparenchymal being the most frequent; leptomeningeal having the worst prognosis, especially in the diffuse form). MRI is the examination of choice for CNS evaluation. Detecting abnormalities within the first 6 months of life was the main risk factor for developing a malignant melanocytic (MM) proliferation, so-called melanoma in childhood according to a recent cohort (12% of patients with CNS abnormalities on MRI develop “melanoma” during childhood) [17–20].

Regarding melanocytic malignancy (MM), the actual incidences are difficult to estimate, with increased risk described in larger lesions, those with more satellite lesions, and those with CNS involvement [21–23]. The overall risk in children with any type of CMN is very low, with an incidence in small lesions of ∼0.1%, and it significantly increases (∼10–15%) in patients with the largest giant lesions (22% of pediatric “melanoma” cases occur in patients with giant CMN), partly due to CNS “melanoma” [24, 25]. Although most primary “melanomas” associated with CMN are cutaneous, it seems that in children the proportion of CNS MM, which are very aggressive, significantly increases, presenting a 100% mortality rate regardless of their location [26].

Although the diagnosis of CMN is primarily clinical, and routine follow-up is conducted through general, neurological, and dermatological physical examination using dermoscopy (sometimes supplemented with other techniques such as ultrasound, confocal microscopy, or MRI), histological and genetic studies play a fundamental role in the investigation of the main complications (MM and NCM), as well as in the search for new, effective, and specific therapeutic options.

This paper focuses on multiple, large, and giant CMN and reviews the latest findings in pathogenesis and genetics, pathology, and new therapies.

## Pathogenesis

Congenital melanocytic lesions are caused by somatic mutations, leading to local clonal expansion of cells with melanocytic differentiation affecting most commonly the skin and/ or leptomeninges. Melanocyte progenitors are totipotential neural crest cells delaminating from the developing neuroectoderm which eventually will form the central nervous system (CNS), during the second month of gestation, and their persistent derivatives, the peripheral nerve-associated Schwann cell precursors (SCP). Nerve-assisted generation of melanocytes from SCPs is an important pathway in delivering melanocytic clones to circumscribed skin areas [27]. Such progenitors may carry a somatic mutation, leading to clonal cell proliferation. The cellular degree of differentiation at the moment of mutation onset can account for the variable histological patterns, sizes, numbers, and spatial distributions of CMN, including segmental and non-segmental types. Multiple or large CMN, for example, may be associated with CNS structural and/or functional abnormalities, and/or a number of different tumors including lipoma, rhabdomyosarcoma, and others [28, 29], supporting an early embryonic onset of a pathogenic mutation [27, 30–32]. The emergence of CMNs from immature neurovascular bundles has also been recognized in pediatric specimens. [33, 34].

The two most frequent genetic alterations found in congenital melanocytic lesions are mutations in the *NRAS* and *BRAF* gene, as initially reported in 2007 by Boris Bastian’s group [35] and later confirmed by Kinsler et al. [36], who studied skin and CNS lesions of 15 patients and found a missense mutation in codon 61 of the *NRAS* gene in the skin and CNS lesions in 12 of them. These mutations were not present in unaffected tissues which were consistent with a post zygotic mosaicism. In 10 patients, the mutation was c.181C > A, p.Q61K, and in 2 patients c.182A > G, p.Q61R. Additionally, loss of heterozygosity was associated with progression to MM in 2 cases. From this study, it was concluded that a postzygotic mutation in *NRAS* is responsible for the majority of cases of multiple CMNs with associated neurological involvement. A subsequent study analyzed a series of patients with large and giant NMCs and compared them with small and medium-sized NMCs. They only found a mutation in *NRAS* in large or giant lesions, while in small and medium-sized nevi this gene mutated in 70% of the cases, and the *BRAF* gene in 30% [37].

However, Salgado et al. [38] studied samples from 66 patients with CMN, 77.3% had a mutation in *NRAS* Q61, and 7.6% had a mutation in *BRAF* V600E. Although the mutation in *NRAS* was found in the majority of giant-, large-, and medium-sized NMCs; *BRAF* was also mutated in 5% of large and 11.4% of giant CMNs. Twenty-four percent of the series had melanocytic lesion involving the CNS, 75% of which had *NRAS* Q61 mutation and 12.5% had *BRAF* V600E mutation. Furthermore, they observed that patients with *BRAF* mutation presented nevi with dispersed or extensive nodules. This has been demonstrated again in a larger series (134 patients) that found the *NRAS* mutation in 68% of patients, the *BRAF* mutation in 7% (70% being multinodular lesions), and in 25% no mutation was found in neither of these 2 genes. Recently, two new mutations involving *RAF1* and *ALK* have been found in 2 patients with giant CMN [39]. Also, Martin SB et al. used RNAseq in 19 patients with no pathogenic mutations in either *NRAS* or *BRAF* and found mosaic *BRAF* fusions in 11 and a mosaic *RAF1* fusion in 1 patient [15].

No higher incidence of MM or neurocutaneous melanocytosis was found based on the genotype. It is believed that mutations in *NRAS* could be more tolerable in early embryogenesis and, at least in part, these could be the reason for the higher prevalence of this mutation. However, the same mutations affecting codon 61 of the *NRAS* gene have been described in a high percentage (15–20%) of conventional (adult) melanomas not associated with CMN, in other tumors, and lesions with different phenotypes [40].

## Molecular pathways and signaling networks

The main genetic alterations described as causing giant CMN involve the RAS/mitogenic activation protein kinase (MAPK)-dependent signaling pathway (RAS/MAPK), responsible for regulating cell proliferation, differentiation, survival, and death.

RAS proteins act as essential mediators in the transformation of extracellular stimuli into intracellular signals depending on their coupling to GDP (inactive form) or GTP (active form). The stimulation of cellular receptors causes the dissociation of GDP from the RAS protein and coupling with GTP, which activates it and promotes the interaction with various effectors such as RAF, MEK, and ERK proteins. These function on a large number of molecules, both cytosolic and intranuclear, and are ultimately responsible for maintaining the cellular life cycle. GTPase activity limits the activation of RAS proteins through conversion of the active form bound to GTP, to the inactive form bound to GDP. The mutations described in giant CMN cause the constitutive activation of the proteins they affect. Posch et al. [41] observed that, although cells with *NRAS* mutation were more sensitive to MEK inhibition, when MEK was inhibited, PI3K signaling became more important for cell survival, so both pathways should be considered relevant.

The PI3K/AKT/mTOR signaling pathway is a signal transduction system that participates in survival and apoptosis, cell growth and proliferation, and cell adhesion and motility, in response to external stimuli. It presents multiple cross-interactions with other signaling pathways, including the RAS/MAPK pathway [42]. PI3K signaling has been shown to be indispensable for maintaining growth in RAS-mutated cell lines both in vitro and in mouse xenografts. Different inhibitors of the PI3K pathway have been tested in in vitro and in vivo models of CMN with encouraging results, alone and in combination with inhibitors of the RAS/MAPK pathway.

## Histopathology

**Fig. 2:**
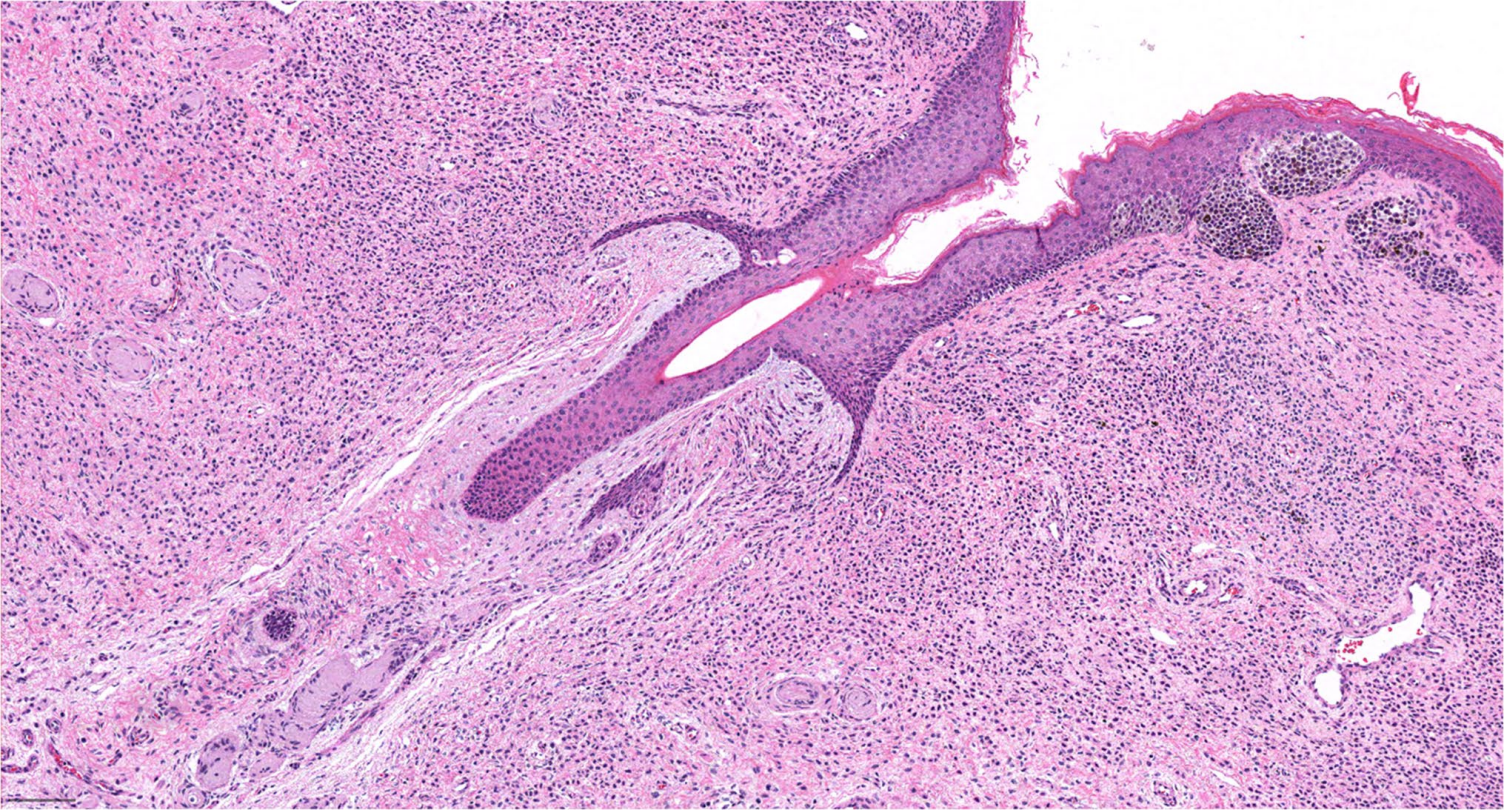
Classical appearance of a congenital melanocytic nevus with a compound architecture (junctional and dermal components), prominent dermal band-like distribution of the lesional cells, infiltration of the skin adnexa and “so-called” neurotization

Melanocytic lesions with congenital features generally have a compound histologic architecture with a prominent band-like pattern; however, many lesions have no junctional component and feature a well-defined Grenz zone in the papillary dermis. The epidermis shows variable atrophy of rete ridges, but papillomatosis or verrucoid changes are relatively common (Fig. 2). In newborns and young children, it is frequent to see a dense population of nevocytes within the deep (reticular) and superficial (papillary) dermis, with only focal junctional involvement that progressively increases as more nevocytes reach the superficial area. In later ages, there is a better-defined junctional dermo-epidermal nevocytic population that gradually decreases in adolescence, leaving an almost non-existent dermo-epidermal junctional component.

**Fig. 3:**
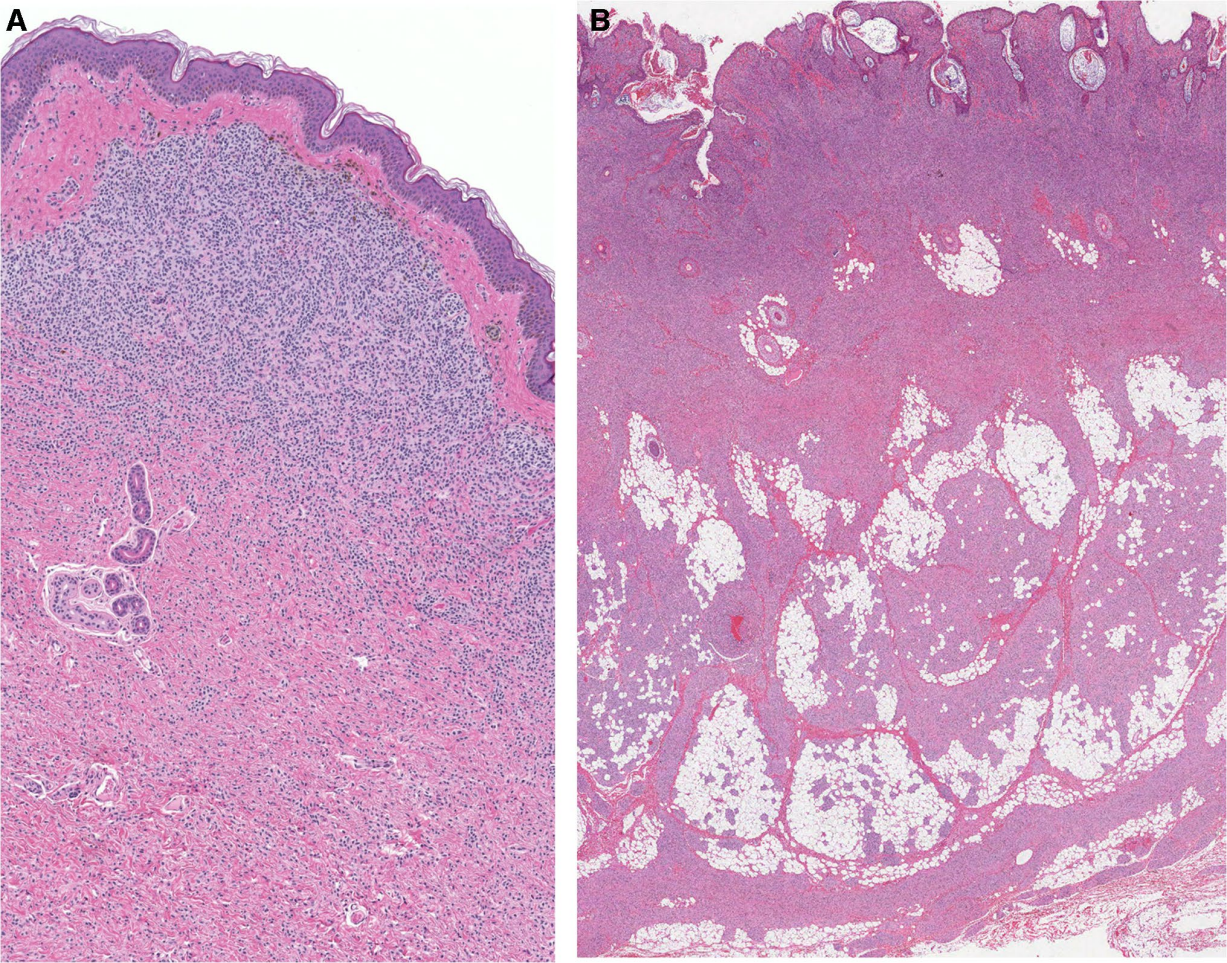
Histologic appearance of congenital melanocytic nevus with a dermal (**A**) or compound (**B**) histologic architecture and prominent band-like pattern. **A** The melanocytic lesion shows no junctional component and a well-defined Grenz zone in the papillary dermis with atrophy of rete ridges. **B** Lesion with prominent papillomatosis (verrucoid features) and nevomelanocytes in the deep subcutaneous tissue/ fascia

**Fig. 4:**
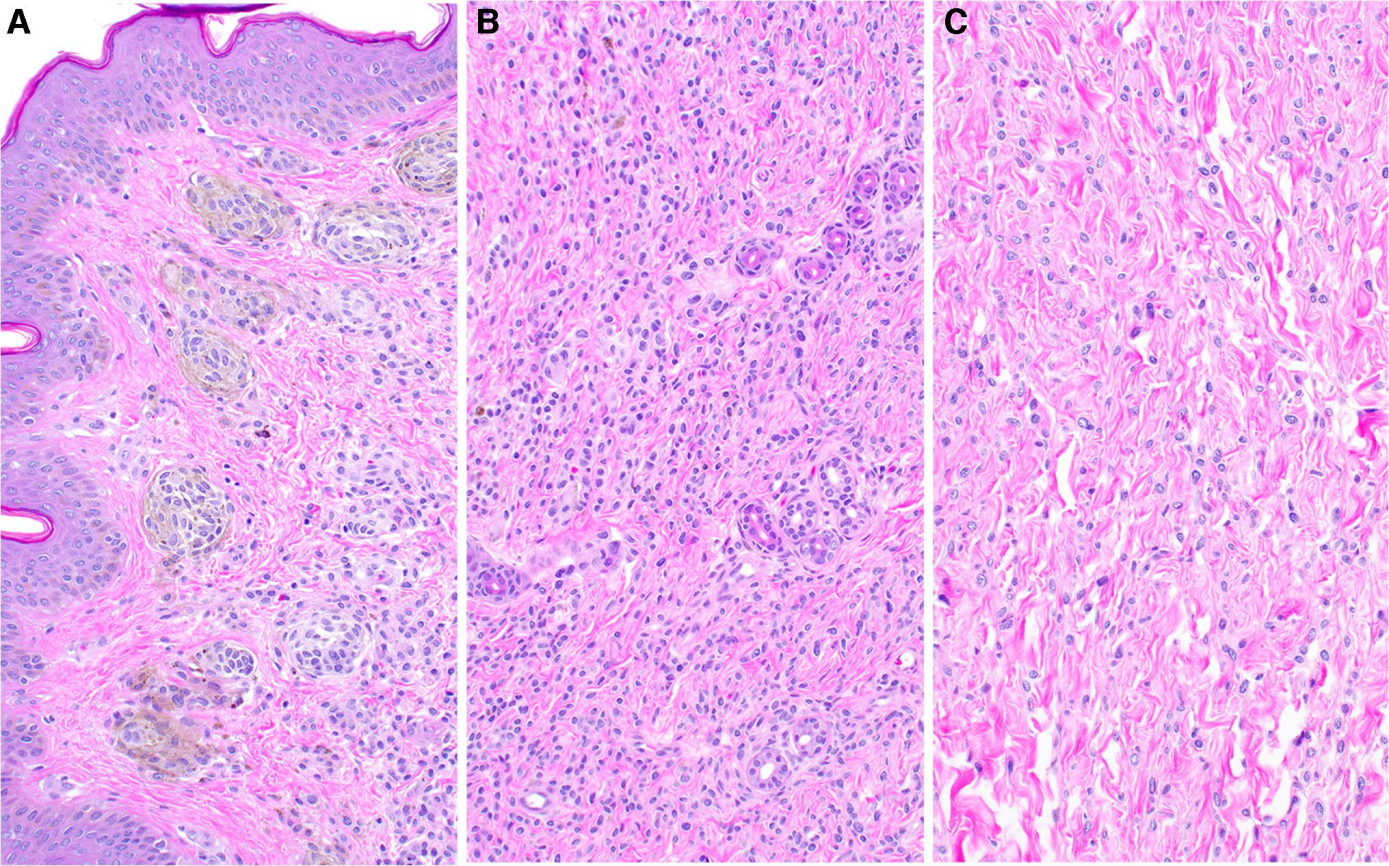
**A** Superficial area of the lesion composed by more mature nevomelanocytes (type A); larger epithelioid cells with abundant cytoplasm and intracytoplasmic melanin. **B** Midway through the nevus, it is composed by compact groups of round cells (type B) which appear as single files between collagen bundles infiltrating adnexal structures. **C** In the deep dermal layers and subcutaneous tissue, the lesional cells are spindly and still immature (type C nevocytes) with a neuroid phenotype (resembling their nerve-sheath based predecessors, the Schwann-cell precursors), poorly defined cytoplasm and limited pigmentation

Histologically, congenital melanocytic nevi (CMN) are characterized by the presence of nevocytes (nevus cells) of variable appearance (Figs. 3 and 4). The lesion in general is present in the deep dermal layers and subcutaneous tissue. In these areas, the lesional cells are spindly and still immature (type C nevocytes) with a neuroid phenotype (resembling their nerve-sheath-based predecessors, the Schwann-cell precursors) [27], poorly defined cytoplasm, and limited pigmentation. A schwannoma-like appearance in the form of Wegner-Meissner or Masson bodies, so-called “neurotization,” is a frequent feature [43]. Midway through these usually thick lesions, compact groups of round cells (type B nevocytes) appear as single files between collagen bundles and intervening lobules of fat, infiltrating adnexal structures and subendothelial spaces. In the more superficial (papillary) dermis, the more mature lesional cells are larger, more epithelioid, with abundant cytoplasm (type A nevocytes), and usually feature intracytoplasmic melanin. Commonly, these superficial nevocytes are surrounded by clusters of heavily pigmented melanophages [44].

Most CMN shows only mild pleomorphism with no significant atypia. However, newborns with large lesions may have impressive pagetoid patterns, atypia, mitoses, and ulceration, not necessarily associated with a malignant clinical progression; therefore, the diagnosis of MM in newborns and young infants should be made with extreme caution [45].

The risk of malignancy, or so-called “melanoma” in the setting of CMN, is a significant concern, particularly in large and giant nevi. Malignant transformation can occur at any age but is more common during childhood and adolescence. Histopathological examination of suspected malignancy in the setting of CMN can be challenging since it frequently does not follow the histologically established criteria for adult (conventional) melanoma. Melanoma in adults, in the classic concept, is more frequently a UV light-induced malignant neoplasm (with the exception of those in acral and “special sites”) which arises as multiple mutations accumulate in (mature) melanocytes. In contrast, malignant melanocytic neoplasia in CMN arises after a much lower number of mutations occurring in nevocytes within a CMN. In addition, recent methylation studies on a cohort of autopsy specimens with CMN and malignant progression found that the neoplasms show a methylation profile different from classic melanoma (manuscript in preparation; [46]).

At birth and in the first months of life, lesions are usually highly cellular, and mitoses are frequent. A difficult and frequent lesion which occurs in large CMNs is the so-called “proliferative nodule” (PN), representing a well-delimited compact aggregate of nevocytes surrounded by conventional nevus areas. Features commonly indicative of malignant behavior in melanocytic lesions of adults such as ulceration, pagetoid spreading, and brisk mitoses are common in proliferative nodules. However, despite the histological resemblance to melanoma, the clinical behavior of this type of lesion is benign [47], leading to difficulties in defining the criteria for the diagnosis of malignancy in this clinical setting.

The following diagnostic criteria favor PNs over melanocytic malignancy in the setting of CMN: symmetry, circumscription, mild to moderate pleomorphism, low to no mitotic activity without deeply located mitoses, evidence of “maturation”, with less pigment and smaller cells in the deeper areas, no necrosis, lack of invasion of surrounding tissue, also referred as “destructive growth” [48].

The usefulness of immunohistochemistry for PRAME, and of analysis of *TERT* promoter and p16 rearrangements to differentiate PNs from melanocytic malignancy in CMN has been recently reported by some groups [49, 50]. Although the studies are still relatively limited and more work is required to establish a firm conclusion, the current opinion is that PRAME immunohistochemistry may have diagnostic value in separating PNs from malignant melanocytic proliferations, although diffuse expression is not specific for malignancy [50]. For this reason, we need to emphasize that the diagnosis of melanoma in pediatric patients with congenital melanocytic nevi requires extreme caution and should be restricted to those instances when there is a combination of an expansile and destructive growth associated with high mitotic activity, necrosis, and ulceration in a patient outside the neonatal group. A particularly useful indicator to support malignancy is the loss of H3K27me3 expression [51]. Also supportive is the finding of multiple abnormalities by comparative genomic hybridization, loss of heterozygosity [36], and amplification of mutated *NRAS* [38]. For lesions without these findings, a descriptive diagnosis acknowledging the limits of predictive accuracy is the most appropriate approach [45].

**Fig. 5:**
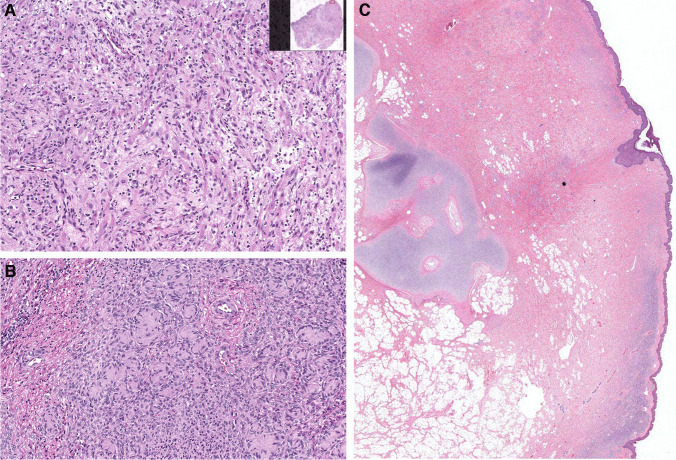
Recapitulating their presumed neural crest origin, nevus cells can show aberrant lines of differentiation. (**A**) CMN with a nodular lesion showing neufibroma-like appearance with few ganglion cells. (**B**) A schwannoma-like pattern in the form of Wegner-Meissner or Masson bodies. (**C**) Represents a CMN with hamartomatous appearance, excess adipose tissue and other mesenchymal elements, including cartilage and aberrant vasculature

It is important to note that congenital melanocytic lesions usually have a malformative and hamartomatous appearance which can vary according to their clinical presentation and age. One example is the large/giant CMN involving the perineal region presenting as a striking tumor composed of nevocytes, termed “bulky perineal nevocytoma” [52, 53]. This is a variant of congenital nevus which may be confused with malignancy. Additionally, recapitulating their presumed neural crest origin, nevus cells can show aberrant lines of differentiation, and some lesions may have excess adipose tissue and other mesenchymal elements, including cartilage and aberrant vasculature (Fig. 5). This may explain why, these lesions may occasionally harbor malignant mesenchymal tumors such as rhabdomyosarcoma, liposarcoma, or peripheral nerve sheath tumors [29, 43].

Another interesting and potentially confusing point is the overlap that exists between CMN and the so-called cutaneous neurocristic hamartomas (CNCH). Both are derived from the neural crests (therefore they are neurocristic), and both have a hamartomatous nature. Well-defined nodular lesions arising independently or within a CMN may fulfill the criteria of CNCH and represent a diagnostic challenge. Some of these CNCH may evolve into melanocytic malignancy, which adds to the difficulties in histopathological analysis. In a case reported by Garrido et al. [54], comparative genomic hybridization revealed an aberration pattern similar to that seen in other PNs.

## Complications

There may be multiple complications associated with CMN. Large/giant CMN usually show mast cell hyperplasia associated with significant pruritus [16, 14, 53, 55]. This may be an important histological feature to be reported because adequate treatment targeting mast cell-induced pruritus can improve the patient’s symptoms and quality of life.

**Fig. 6:**
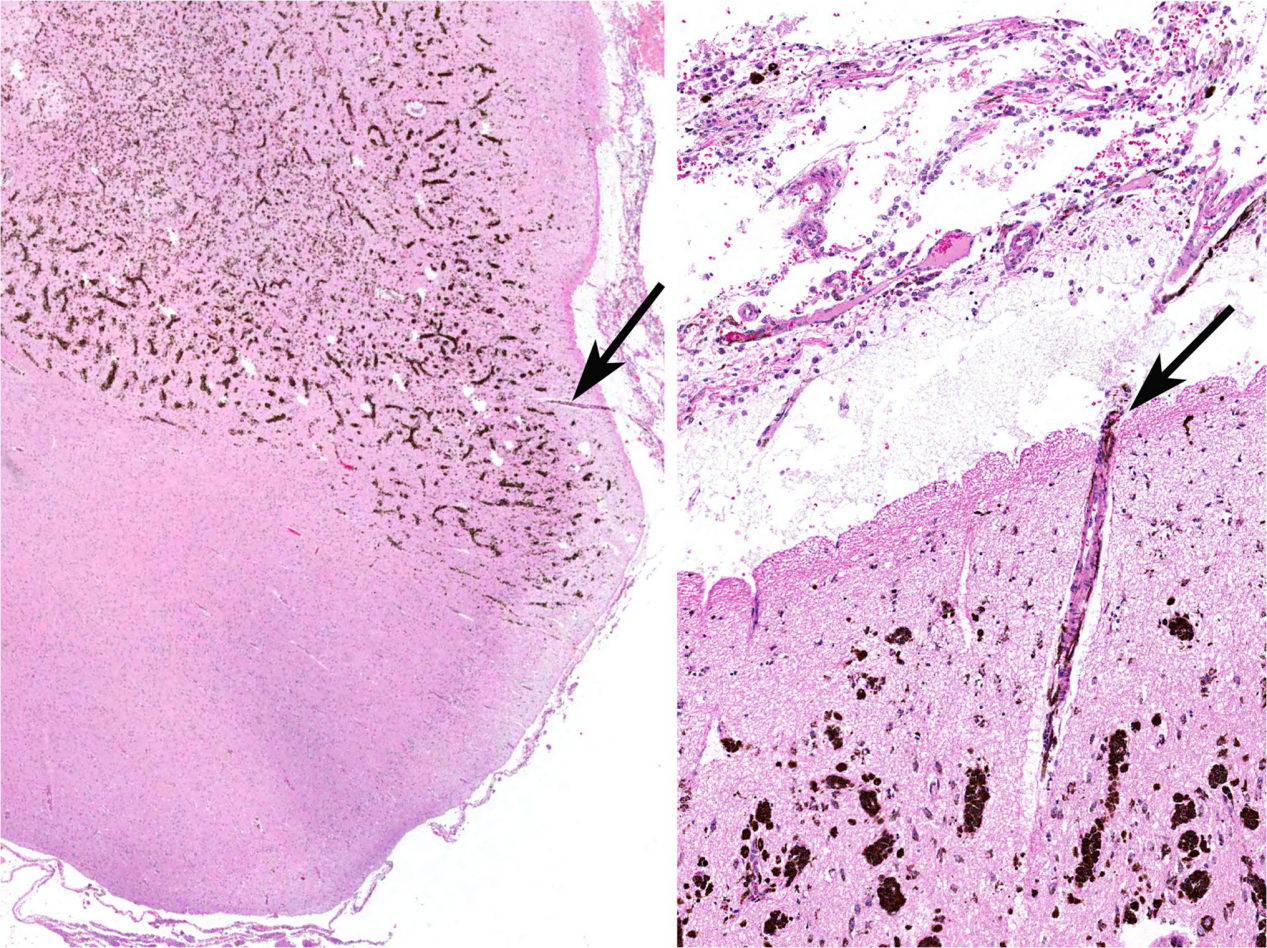
Representative images of a 9 year-old patient with NCM. Left: low power view of the hippocampus heavily pigmented nevomelanocytes infiltrating the central nervous system parenchyma in a predominantly perivascular pattern. Right: slightly higher magnification showing the same blood vessel with its perivascular Virchow-Robin space populated by invading nevomelanocytes which infiltrate the brain parenchyma. Observe the overlying leptomeninges with the heavily pigmented proliferating nevomelanocytes. The arrows in both frames point to the same blood vessel

One of the most feared and serious complications associated with large/giant congenital melanocytic nevi is the development of central nervous system involvement, a syndrome referred to as neurocutaneous melanocytosis (NCM), in which the meninges and/or multiple foci of the central nervous system develop melanocytic proliferations (Fig. 6) associated with postzygotic somatic mutations affecting *NRAS*, which represent the first hit in a multistep oncogenetic process. Amplification of mutated *NRAS* also leads to an aggressive form of NCM with wide dissemination of congenital melanocytic malignancy within the CNS [12, 38].

Multiple cases of “congenital malignant melanomas” are available in the literature. A remarkable lethal example reported by us [38] involved a newborn with the full spectrum of neurocutaneous melanocytosis, including a CMN, satellite lesions, and extensive CNS involvement. In this case, there was a five-fold increase in *NRAS* expression in the malignant melanocytic cell line compared to normal melanocytes. Incidentally, the amplification of a mutated *NRAS* gene has been reported also by Dr. de la Fouchardiere’s group in an adult who developed malignant melanoma arising in a trichroblastoma [56]. Other recent example of malignant melanocytic neoplasia arising in-utero within CMN were recently described [57] in which oncogenic ZEB2::ALK fusion genes were identified in both, the nevus and the malignant cells.

There are also reports of non-melanocytic malignant tumors arising in the setting of CMN. Hendrickson and Ross collected several of these cases [58]. Among these malignancies, the most common appears to be rhabdomyosarcoma, with several reports available in the literature [59–62]. Additional types of tumors arising in the setting of CMN, include neurocristic tumors such as schwannomas, neurofibromas, and mixed malignant peripheral neuroectodermal tumors, and non neurocristic types such as liposarcomas and dermatofibrosarcoma protuberans.

## Treatment

### Current options

For tissue fragility-induced erosions in CMN, conservative management with dressings usually results in good healing. Chronic pruritus can be managed with emollients and topical corticosteroids in some cases. However, it can be severe and refractory, impacting the quality of life and requiring surgical excision of the affected area, as well as lesions with overgrowth and folds in which hygiene may be difficulted, affecting significantly the quality of life.

Regarding the treatment of cutaneous lesions, there are currently two options in routine clinical practice. On one hand, observation is a valid option, as treatment is not necessary for lesions without complications. On the other hand, surgery, which involves different techniques of varying complexity (such as the use of tissue expanders or grafting), depending on the size and characteristics of the lesions is a frequently recommended option. However, some aspects of surgery are controversial, such as the reduction of melanoma risk and aesthetic improvement. For symptomatic lesions (e.g., with pruritus, refractory erosions, or exophytic tissue), or patients with psychological or aesthetic concerns who desire intervention, surgery is an important consideration. Currently, the decision-making process is advocated to be personalized in collaboration with the patient and their family, explaining the options along with their pros and cons.

Regarding medical treatments tested in isolated cases, a 7-year-old patient with giant verrucous CMN with AKAP9-BRAF fusion was treated with trametinib, which improved pain, pruritus, and the exophytic component [14]. Also, recently, two cases of patients with BRAF fusion described rapid improvement in protruding tissue, inflammation, and pruritus with the same treatment [15].

There are no specific treatments for complications. CMN patients who develop MM are treated similarly to other melanoma patients, based on the stage and mutations found. Of note, activating *NRAS* mutations (the most common in CMN) is believed to activate both the MAPK and PI3K pathways in *NRAS*-mutated melanoma cells [41]. No clinical trials have been conducted specifically in CMN patients, and there is limited solid data on treating MM associated with CMN. Therapeutic options should be discussed in a multidisciplinary committee, especially for advanced cases, primary CNS MM, or associated with large/multiple CMN. MM on giant CMN exhibit more aggressive behavior, particularly in childhood, and primary CNS MM are generally fatal within 6 months of diagnosis [26]. In the latter cases, ventriculoperitoneal shunting, systemic corticosteroid therapy, and radiotherapy can be used for symptomatic control. For patients with neurocutaneous melanocytosis requiring treatment, options are similar: surgical excision of symptomatic tumor lesions when feasible, ventriculoperitoneal shunting for intracranial hypertension, or anticonvulsants for epileptic seizures [26].

Regarding recent treatments tested in isolated cases, MEK inhibitors are noteworthy. In pediatric patients with CNS MM (*NRAS* mutated), trametinib administration improved symptoms, although the disease ultimately progressed [63]. For NCM, MEK inhibition as compassionate treatment in a child with progressive symptomatic NCM (also *NRAS* mutated) resulted in a decrease in lesion proliferation, unfortunately followed by death a few days later without clinical improvement [64].

### Experimental therapies

Currently, novel pharmacological treatments for CMN are under investigation.

Table 1 summarizes the main results of some experimental treatments tested in recent years in various cell or murine models.

**Table 1:** Summary of the results of experimental treatments for congenital melanocytic lesions

MEK and PI3K/mTOR pathway inhibitors			
Treatment	Model	Result	Reference
Alpelisib (PI3K pathway inhibitor)	Tissue explant cultures obtained from patients with CMNG	Decrease in nevomelanocytes	Tomás-Velázquez A et al. J Eur Acad Dermatol Venereol [65]
Trametinib (MEK inhibitor)	CMN cell culture (BRAF fusion)	Inhibition of MAPK pathway hyperactivation	Martin SB et al. J Invest Dermatol [15]
Various MEK and AKT inhibitors	Nevospheres, explant cultures, and xenografts in mice (NRAS mutated)	Decrease in cell viability and proliferation	Rouille T et al. J Invest Dermatol [66]
Omipalisib (PI3K and mTOR inhibitor) alone and in combination with MEK162 (MEK inhibitor)	CMN cell culture	Suppresses colony formation and induces cell death (autophagy), synergistic activity in combined treatment	Basu D et al. Cancer Genomics Proteomics [67]
Selumetinib (MEK inhibitor)	Murine model of CMN with CNS involvement (NRASQ61K activated and Wnt signaling)	Suppression of nevomelanocytic development in skin and CNS	Pawlikowski JS et al. J Invest Dermatol [68]
MEK, PI3K/mTOR, and Other Pathway Inhibitors			
Treatment	Model	Result	Reference
MEK, PI3K, and c-KIT inhibitors and SADBE-based immunotherapy (squaric acid dibutyl ester-based immunotherapy)	Murine models with CMNG xenografts (among others)	Lesion regression with all four treatments, SADBE through an innate immunity-mediated inflammatory response	Choi YS et al. Cell [69]
Histone deacetylase inhibitors (HDACi)	CMNG cell cultures	Suppression of MITF expression, senescence, and cell death (necrosis)	Basu D. et al. Melanoma Res. [70]
MEK, PI3K/mTOR, and IGF-1R inhibitors	Murine model of CNS involvement	Inhibition of cell growth, synergistic with combined PI3K/mTOR and IGF-1R inhibition	Ruan Y. et al. Neuro Oncol [71]

Some of these treatments may become non-surgical alternatives for CMN. Clinical trials are needed to investigate the efficacy and safety of them as well as their long-term outcomes.
